# Neural Basis of Two Kinds of Social Influence: Obedience and Conformity

**DOI:** 10.3389/fnhum.2016.00051

**Published:** 2016-02-22

**Authors:** Ying Xie, Mingliang Chen, Hongxia Lai, Wuke Zhang, Zhen Zhao, Ch. Mahmood Anwar

**Affiliations:** ^1^School of Management, Zhejiang UniversityHangzhou, China; ^2^Scholars IndexJersey City, NJ, USA

**Keywords:** neural basis, conformity, obedience, social influence, event-related potentials

## Abstract

Event-related potentials (ERPs) were used in this study to explore the neural mechanism of obedience and conformity on the model of online book purchasing. Participants were asked to decide as quickly as possible whether to buy a book based on limited information including its title, keywords and number of positive and negative reviews. Obedience was induced by forcing participants to buy books which received mostly negative reviews. In contrast, conformity was aroused by majority influence (caused by positive and negative comments). P3 and N2, two kinds of ERP components related to social cognitive process, were measured and recorded with electroencephalogram (EEG) test. The results show that compared with conformity decisions, obedience decisions induced greater cognitive conflicts. In ERP measurements, greater amplitudes of N2 component were observed in the context of obedience. However, consistency level did not make a difference on P3 peak latency for both conformity and obedience. This shows that classification process is implicit in both conformity and obedience decision-making. In addition, for both conformity and obedience decisions, augmented P3 was observed when the reviews consistency (either negative or positive) was higher.

## Introduction

Obedience and conformity are two kinds of social influences when people change attitude or behavior under the influence of the views of others. The term “obedience” refers to direct requests from an authority figure to one or more persons (Nail et al., 2000). It is a particular kind of response (i.e., acquiescence) to a particular kind of communication (i.e., a request) (Cialdini and Goldstein, 2004). Obedience is common in life. For instance, children must follow what their parents tell them to do, employees have to obey the orders of their boss and as an extreme example, soldiers have to absolutely follow the instructions of their leaders in the army. Conformity behavior describes various social and economic situations in which individuals are strongly influenced by the decisions of others (Asch, 1956), such as in financial investment, technology adoption, firms’ strategic decisions, political voting, and dining and fashion trends.

A considerable body of literature exists on conformity. Sherif (1935) demonstrated conformity in group members’ judgments of ambiguous perceptual stimuli using autokinetic illusion. Asch (1952) reported a curvilinear relationship between group size and conformity in a group pressure experiment. Some scholars have also used neuroscience tools to explore the neural and psychological basis of conformity (Chen et al., 2010).

In obedience research, however, studies have not completely got rid of Milgram (1974) classic shock experiment paradigm yet. In Milgram’s research, one naive subject and one victim, who should be an accomplice, are required in each experiment. The naive subject was always the teacher, and the learner (an accomplice) was trapped into an “electric chair”. The teacher (the real participant) was taken to a desk with an instrument panel ranging from 15 to 450 volts. Than the teacher was told to administer a greater shock to the learner each time when he gives a wrong response. The victim (the accomplice) was trained to play the role, and no actual electric wave was applied. As mistakes made by victim increased, more intense shocks were required to be applied by the teacher. In the meantime, internal resistance of victim became stronger, and at a certain point the “teacher” refused to go on with the experiment. Behavior prior to this rupture was considered “obedience”, in that the subject complied with the commands of the experimenter. However, there was a problem laid in this study design: participants were put into choice dilemma involving moral issue. To follow the authority order, they had to break inner moral rules. Administering electric shocks to another person, especially on a voltage more than human tolerance range, is inhuman and beyond the moral boundary. Questions and discussion about this paradigm have never ceased (Miller, 1986; Blass, 2009). Many scholars have made improvements on Milgram’s paradigm. For instance, Mixon (1972) simulated destructive obedience through the use of role-playing, whereas, Burger (2009) ended the study at 150 volts. Zeigler-Hill et al. (2013) introduced noise blasts as substitute for electric shocks. These were all great works but they still have not jumped out of the shadow of Milgram’s paradigm and still involve some moral decision-making.

As we mentioned above, obedience and conformity are different from each other though they have something in common (independance and submission). Theoretically, conformism can be delineated as change in thinking, feelings, acting due to external pressure, imaginary or real, whereas, obedience is the manifested modification in behavior carried out as a result of authority instructions. However, conformism is directed psychological state that can be disposed towards obedience. It is not hidden that role of conformism and obedience is very crucial in social life and without them social life could become completely disordered and confused. Due to the limitation of experimental paradigm, not many studies on obedience have been conducted compared to studies on conformity. Psychological process underlying obedience remains unclear. Neural basis of both obedience and conformity also remain a mystery.

In this study, we aimed to explore the similarities and differences in the neural mechanisms of conformity and obedience by stimulating consumers to purchase books online. Books are experience products, associated with high degree of involvement and low price (Chen, 2007). The purchase decision is easily affected by the opinion of others or the authority. Furthermore, the leading factors for evoking conformity and obedience behavior in real book-purchasing situation can be simulated easily in the laboratory. The study was divided into two parts: conformity part and obedience part. In the conformity part, participants were asked to make purchasing decisions on the basis of positive and negative reviews appearing in the stimuli picture. Generally, people tend to choose the products most often praised by others (Cohen and Golden, 1972). In the obedience part, participants were requested to buy books that received mostly negative reviews of previous customers. It is also worth mentioning that researchers studied conformism processes in the context of opinion dynamics (see Friedkin and Johnsen, 1990; Javarone, 2014). The identification of endogenous conformity values of people is critical to enable many remarkable applications that leverage opinion dynamics.

The measurement of event-related potentials (ERPs) is used as a “magnifying glass” to observe mental processes without asking consumers directly for their thoughts, memories, evaluations, or decision-making strategies. ERPs thus can provide access to the otherwise hidden information (Plassmann et al., 2012). The components N2 and P3 of the waveform are considered to be social cognitive related (Fabiani et al., 2000). Nieuwenhuis et al. (2003) demonstrated that N2 is the reflection of cognition conflict, with the amplitude increasing when individual faces greater cognition conflict. Yang et al. (2007) also believe that perceived conflict and perception difficulty will induce larger N2. P3 is considered to reflect neuroelectric activity related to cognitive processes (Polich and Kok, 1995). The amplitude of P3 is influenced by task difficulty and confidence. The more difficult is the task, or the less confident the judgment is, the smaller is P300 amplitude (Johnson and Donchin, 1978).

## Materials and Methods

### Participants

Fourteen healthy students studying management as major subject in Zhejiang University of China voluntarily participated in this ERP experiment. Their average age was 24.14 (range 22–26), five of them were female. They were all right handed, had normal vision or normal corrected visual acuity and had no history of neurological or mental diseases. Before commencing the study, all subjects signed the “Zhejiang University neural science laboratory test informed book” and “the experimental process confirmation letter”.

### Materials

Stimulus pictures contain three main items: book title keywords, number of positive reviews, and number of negative reviews. Fourty five books were chosen from Amazon and Dangdang, two popular online book sellers in China, in the field of customer relationship management (CRM). These CRM books appeal to the students of management. The title keywords of each book were limited to four Chinese characters, in order to eliminate the title influence on subjects. The total number of book reviews ranged between 400 and 500. The percentages of the number of positive reviews in the total number of reviews were fixed at 0, 25, 50, 75, and 100%, corresponding to absolute negative review, relatively negative review, inconsistent review, relatively positive review, and absolute positive review, respectively. Thus, each book was allocated into one of the five categories based on the reviews consistency. There were 225 stimuli pictures created in total. It should be pointed out that inconsistent review stimulus is presented to make the participants feel the experiment to be authentic, but data of this group were excluded from the final data analysis.

### Procedure

Participants sat on a comfortable sofa located in a shielded room and were instructed to avoid frequently blinking or moving their eyes. The stimuli (white on a black background) were presented continuously and randomly in sequence in the center of a computer screen, with a visual angle of 2.58° × 2.4°. In each trail: picture “+” was first presented for 200 ms, followed by 300 ms of stimulus interval and 1400 ms of stimulus picture that contained book title keywords, number of positive reviews and number of negative reviews. Subjects should have made a decision as quickly as possible during this 1400 ms. Regardless of whether they did or did not have enough time to make their choice, the sequence was followed by 500 ms of black background which indicated the end of a trail. Although all confounding variables have been controlled in order to overcome external influences, the latter may in principle be present due to the participants’ hobbies and/or inclinations. Thus, further investigations will be required to understand if, and under which extent, external influences may affect the achieved outcomes.

The whole experiment consisted of two parts. Stimulus materials were the same in both segments, all including two blocks. There was a 3–5 min break between two blocks, and the participants could have a rest for 10–15 min between two segments. In part 1, participants were told to decide by themselves according to the information in the picture. In part 2, participants were requested to buy books which had larger number of negative reviews compared to positive ones. By following the similar procedure like part 1, obedience was induced by forcing participants to buy the book with lots of negative comments. For books with inconsistent reviews, the subjects were free to make any decision. We followed the pattern i.e., conformity than obedience because “those that conform tend to be obedient and compliant” (Constable et al., 1999).

Before the start of experiment, participants were asked to read the experiment instructions. At the same time, participants were required to complete a practice to make sure that they understood the tasks and familiarized themselves with the experiment program. Participants should have responded to the stimulus material within 1400 ms using left button to buy and right button not to buy. Trails in which no button was pressed were regarded as invalid tests, and the data from these trails were not analyzed.

### Electroencephalogram Recording and Analysis

Neuroscan Synamp2 Amplifier (Scan 4.3.1, Neurosoft Labs, Inc., Sterling, VA, USA) was used to obtain continuous data of the electroencephalogram (EEG), using an electrode cap with 64 Ag/AgCl electrodes mounted according to the extended international 10–20 system and referenced to linked mastoids. Vertical and horizontal EEGs were recorded with two pairs of electrodes, one placed above and below the left eye, and the other 10 mm from the lateral canthi. Electrode impedance was maintained below 10 Ω throughout the experiment.

The recording started 100 ms (used as the baseline) before the onset of each picture, and ended 800 ms after the presentation. Electrooculogram artifacts were corrected using the method proposed by Semlitsch et al. (1986). Trails contaminated by amplifier clipping, bursts of electromyographic activity, or peak-to-peak deflection exceeding ±80 μV were excluded from the analysis. The average ERPs were digitally filtered with a low-pass filter at 30 Hz (24 dB = octave). Within-subjects design of Analysis of Variance (ANOVA) with repeated-measure was used to explore the neural mechanisms of subjects in different situations.

## Results

### Behavioral Data

Conformity rate is the ratio of subjects who made conformity decision according to the number of positive and negative reviews (decide to buy a book under relatively positive and absolute positive conditions and not to buy a book under relatively negative and absolutely negative conditions) in part 1. It was calculated according to the following formula: Conformity rate = conformity decision/(conformity decision + inconformity decision). Obedience rate stands for the ratio of participants who followed what the examiner told them to do (decide to buy a book under relatively negative and absolute negative conditions and not to buy a book under relatively positive and absolute positive conditions). Thus, it was calculated according to the following formula: Obedience rate = obedient decision/(obedient decision + disobeying decision). Response times (RTs) refer to the time periods from the moment the picture was presented to the moment the decision was made. It indicates the shortest time one need to make the purchase decision. Table 1 illustrates the behavioral data obtained for conformity and obedience.

**Table 1:** **Conformity/obedience rate and response times (RTs)**.

Condition	Conformity/obedience rate	Conformity/obedience (*SD*)	RTs	RTs (*SD*)
Conformity	93.78	6.67	637.02	81.32
Obedience	95.71	3.58	678.62	88.12

As can be seen from the Table 1, the total conformity rate (mean = 93.78, *SD* = 6.67) is lower than the total obedience rate (mean = 95.71, *SD* = 3.58). However, *t*-test shows no significant difference between conformity and obedience (*t* = −0.912, *p* = 0.378). RTs for conformity were shorter than that for obedience. Still, no significant difference were found between the two groups (*t* = −1.343, *p* = 0.202).

Due to the similarity of review consistency, we further sort the four contexts into two groups: absolutely consistent context (absolutely positive trails and absolutely negative trails) and relatively consistent context (relatively positive trails and relatively negative trails). Data for these two categories of review consistency are given in Table 2.

**Table 2:** **Conformity and obedience data of two categories of review consistency**.

	Conformity				Obedience			
Condition	Rate (%)	*SD*	RTs	RTs (*SD*)	Rate (%)	*SD*	RTs	RTs (*SD*)
Absolute consistent review	97.53	3.76	580.24	82.27	97.07	1.71	631.35	97.21
Relative consistent review	90.00	10.09	693.80	87.88	94.36	6.93	725.89	88.96

It is clear from the table that review consistency affected both conformity and obedience. In conformity section, conformity rate is higher in the absolutely consistent context (97.53%) than that in the relatively consistent context (90.00%; *t* = 3.853, *p* = 0.002). RTs are shorter in the absolutely consistent context (580.24 ms) than that in the relatively consistent context (693.80 ms; *t* = −8.438, *p* < 0.001). In obedience section, the obedience rate is also higher in the absolutely consistent context (97.07%) compared to the relatively consistent context (94.36%; *t* = −8.438, *p* < 0.001). RTs are shorter in the absolutely consistent context (631.35 ms) than that in the relatively consistent context (725.89 ms; *t* = −5.841, *p* < 0.001).

### Event-Related Potential Data

#### Main Effect of Social Influence Type

**Figure 1:**
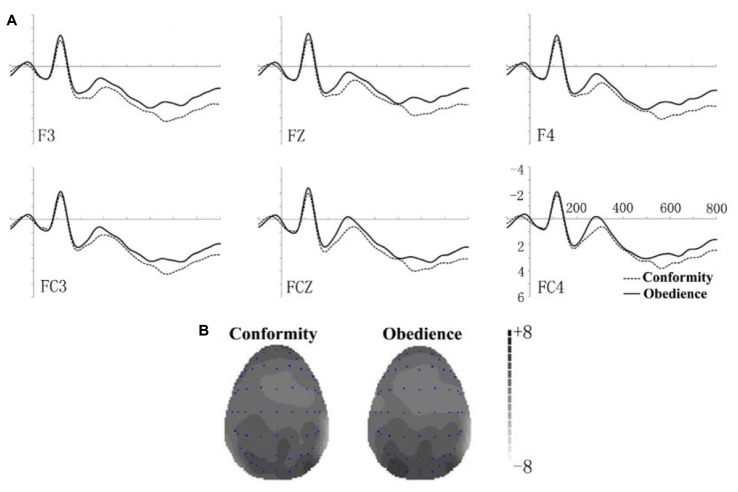
**Event-related potential (ERP) raw waveforms at six electrodes for obedience (light lines) and conformity (dotted lines).** For details regarding Figures **1A,B** please see text.

According to the known distribution of N2 (Yuan et al., 2007), six electrodes (F3, FZ, F4, FC3, FCZ, and FC4) were selected as representative sites. N2 amplitudes in the 240–310 ms time windows were analyzed. Figure 1A gives the overall picture of grand-averaged ERP waveforms for conformity and obedience at different sites (F3, FZ, F4, FC3, FCZ, FC4). N2 amplitudes were larger in the obedience trials (mean = 0.5809 μV, *SD* = 2.8503) and that in conformity trials (mean = 1.4772 μV, *SD* = 2.3421). We performed a 2 (decision type: conformity, obedience) × 6 (electrode site: F3, FZ, F4, FC3, FCZ, FC4) within-subjects repeated measure ANOVA on ERP amplitudes. The result implies that the main effect of social influence type was significant (*F_(1,13)_* = 5.353, *p* = 0.038). Main effect of electrode site was significant (*F_(5,65)_* = 4.782, *p* = 0.014). The interaction between social influence type and electrode site was not significant (*F_(5,65)_* = 0.824, *p* = 0.478). Topographic maps of the maximal amplitudes of N2 (280 ms) are presented in Figure 1B. N2 in the obedience section was more remarkable than that in the conformity section, with almost all frontal and frontal-central areas covered by higher negative potential.

#### Main Effect of Review Consistency

According to the distribution of P3 (Jones et al., 2012), electrode points C3, CZ, C4, CP3, CPZ, CP4, P3, PZ, P4 were chosen for statistical analysis. Relatively positive and relatively negative reviews were regarded as relatively consistent reviews. Similarly, absolutely positive and absolutely negative reviews were regarded as absolutely consistent reviews.

**Figure 2:**
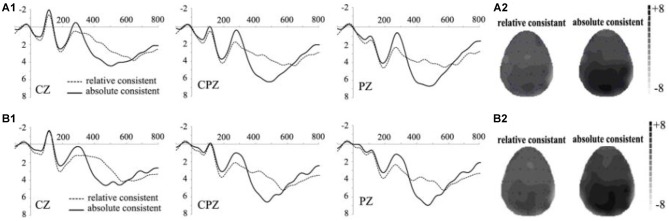
**Grand-averaged ERP waveforms.** For details regarding Figures **2A1,A2,B1,B2** please see text.

Figure 2A1 shows grand-averaged ERP waveforms evoked by absolutely consistent reviews and relatively consistent reviews in the conformity trails at CZ, CPZ, PZ. Absolutely consistent group (mean = 4.500 μV, *SD* = 2.531) yielded larger P3 than relatively consistent group (mean = 3.262 μV, *SD* = 2.589, *p* < 0.001). We performed a 2 (consistency level: absolutely consistent reviews, relatively consistent reviews) × 9 (electrode sites: C3, CZ, C4, CP3, CPZ, CP4, P3, PZ, P4) within-subjects repeated measure ANOVA on P3. Main effect of review consistency was evident (*F_(1,13)_* = 16.038, *p* = 0.001), the effect of electrode site was also remarkable (*F_(8,104)_* = 7.461, *p* = 0.001). The interaction between review consistency and electrode site was not significant (*F_(8,104)_* = 0.682, *p* = 0.557). Topographic maps of the maximal amplitudes of P3 (450 ms) are presented in Figure 2B1. The P3 in the absolutely consistent trials was more remarkable compared to that in the relatively consistent trials, with almost all central, central-parietal and parietal areas covered by higher positive potential.

Figure 2A2 shows grand-averaged ERP waveforms evoked by absolutely consistent reviews and relatively consistent reviews in the obedience trails at CZ, CPZ, PZ. We performed a 2 (consistency level: absolutely consistent reviews, relatively consistent reviews) × 9 (electrode sites: C3, CZ, C4, CP3, CPZ, CP4, P3, PZ, P4) within-subjects repeated measure ANOVA on P3. Absolutely consistent group (mean = 5.098 μV, *SD* = 2.325) yielded larger P3 than relatively consistent group (mean = 3.070 μV, *SD* = 2.952). Main effect of review consistency was evident (*F_(1,13)_* = 31.040, *p* = 0.001), the effect of electrode site was also remarkable (*F_(8,104)_* = 11.554, *p* = 0.001). The interaction between review consistency and electrode site was significant (*F_(8,104)_* = 2.379, *p* = 0.021). Topographic maps of the maximal amplitudes of P3 (450 ms) are presented in Figure 2B2. The P3 in the absolutely consistent trials was more remarkable compared to the relatively consistent trials, with almost all central, central-parietal and parietal areas covered by higher positive potential.

## Discussion

This study aimed to explore neural activities associated with conformity and obedience and further investigate inner neural mechanisms in different situations where these social influences play role. Online book purchasing context was used as a model in this study.

### Behavioral Differences

Total conformity rate observed in this study did not differ significantly from the total obedience rate. This implies that subjects are easily influenced by the majority opinion and the authority command, thus leading to no significant influence of conformity and obedience on the decision-making. There was also no significant difference between the RTs, i.e., the time needed to make decision, in conformity and obedience trials, further proving that majority opinion and authority command played the same important influence on the participants. Consistency level has significant effect on conformity (Burnkrant and Cousinesu, 1975; Pincus and Waters, 1977; Huang and Chen, 2006). Our results indicated that the conformity rate is much higher in the absolutely consistent trails comparing to the relatively consistent trails. Congruence exists between our conclusion and the published data: more subjects choose to follow the majority when the consistency level was high (Burnkrant and Cousinesu, 1975; Pincus and Waters, 1977; Huang and Chen, 2006; Chen, 2007; Chen et al., 2009). Meanwhile, our data indicate that the conformity RTs are much shorter when consistency level is high. It supported our point of view in another perspective, namely that high consistency helped participants to make the conformity decisions. Consistency level also had an impact on obedience. Obedience rate was much higher in the absolutely consistent trails compared to the relatively consistent trails. Obedience RTs in the absolutely consistent contexts were significantly shorter than in the relatively consistent contexts. This may be attributed to the fact that consistency level largely relates to classification. High consistency reduces the difficulty of the decision-making, and people need less time to make a decision.

### Main Effects of Social Influence on N2

N2 reflects the early (250–300 ms) decision-making process (Bekker et al., 2005; Clayson and Larson, 2011) and is distributed in the medial frontal brain areas (Clayson and Larson, 2013). In our experiments, peak latency is evoked at approximately 280 ms after the presence of the picture. Flostein and Van Petten (2008) hold the view that N2, which is generated from anterior cingulate, is bound up with conflict detection (Clayson and Larson, 2011; Buzzell et al., 2014). The conflict detection and conflict monitoring role of anterior cingulate has also been confirmed by many other researches like Carter et al. (2000), Veen and Carter (2002), Sanfey et al. (2003), and Botvinick et al. (2004). Nieuwenhuis et al. (2003) brought out the idea that N2 reflects conflicts, its amplitude becomes higher when conflict aggravates and the amplitude of N2 positively correlates with the reaction time. In our experiment, amplitudes of N2 for obedience (mean = 0.5809 μV, *SD* = 2.8503) were significantly larger than for conformity (mean = 1.4772 μV, *SD* = 2.3421, *p* = 0.038). It implies that obedience arouses greater cognitive conflict. It can be explained by cognitive dissonance theory: when obedient decision contradicts to subjects’ cognition, balance of cognitive factors is destroyed, and mental maladjustment occurs. Previous studies have established that N2 has a positive relationship with cognitive conflicts (Yang et al., 2007; Wang et al., 2010). Obedience is associated with excessive conflicts. Therefore, higher N2 amplitudes are observed in the contexts of obedience comparing to conformity. Moreover, although the task is fairly clear in the obedient situations, subjects need to adjust their cognitive factors to fulfill it. Comparatively speaking, greater efforts must be paid to the obedient decision rather than to the conformity decision. Amplitudes of N2 positively correlate with the cognitive conflict and perception of task difficulty. Consequently, amplitudes of N2 for the obedience were significantly higher than for conformity.

### Main Effects of Consistency on P3

Remarkable P3 waves which vary according to the consistency levels were elicited in both conformity trials and obedience trials. Maximum amplitudes were observed at approximately 450 ms. Amplitude of P3 in the absolutely consistent review situations is much larger than in the relatively consistent review situations and inconsistent situations. A considerable body of literature suggests that comparing to inconsistent reviews, the consistent reviews would attract more supporters (Burnkrant and Cousinesu, 1975; Pincus and Waters, 1977; Weiner, 2000). P3 is an effective index for the core information processing in brain (Palmera et al., 1994). Recent studies have shown that amplitude of P3 is related to the difficulty of decision making (Vallesi, 2011), with greater difficulties arising smaller amplitudes. So, in the absolutely consistent situations, participants can easily follow the consensus groups. Decision-making difficulty is smaller than that taking place under the relatively consistent reviews conditions. Cutmore and Muckert (1998) put forward the idea that the smaller P3 emerges when subjects face greater difficulty in sorting stimulus or lacking confidence to decide. In our experiments, in the absolutely consistent situations, the participants faced clear judgment discrepancy, the sorting stimulus was easy, the participants were determined and confident, and the P3 amplitudes were large. In the relatively consistent situations, the participants faced obscure judgment discrepancy, the sorting stimulus was difficult, the participants were uncertain and not confident. It is worth mentioning that consistency level did not influence the P3 peak latency between conformity decision and obedience decision. This reveals that classification process is implicit in both conformity and obedience. Consistency level may affect conformity and obedience decisions in a similar neuropsychological pattern.

## Conclusion

Event-related brain potentials (ERPs) were used in this study to explore the neural mechanisms of conformity and obedience on the model of online book purchasing. Participants were asked to make a decision, as quickly as possible, whether to buy a book based on the limited information which included book’s title keywords and the number of positive and negative reviews. Conformity was aroused by the majority influence (caused by positive and negative comments). Obedience was induced by forcing participants to buy the book with lots of negative comments. P3 and N2, two kinds of ERP components which are assumed to be social cognitive related, were recorded and studied in this study. Even though behavioral data displayed no remarkable differences between conformity decisions and obedience decisions, ERP results suggest that obedience triggered bigger cognitive conflicts than conformity. On the surface, the subjects were easily influenced by both majority opinion and authority command. Deep inside, however, they were more struggling when making the obedience decisions. In the ERP, greater amplitudes of N2 component were observed in the context of obedience. Consistency level did not make a difference on P3 peak latency for both conformity and obedience, which reveals that a classification process is implicit in both decision types (i.e., conformity and obedience). In addition, for both conformity and obedience decisions, the augmented P3 was observed in the absolutely consistent review situations compared to the relatively consistent review situations.

## Limitations and Future Directions

This study has a number of limitations. The simulation of online book purchase used in this study is rather simple and based on significantly reduced amount of information provided. Measures should be taken to explore better ways to simulate the online purchasing. It might be possible to introduce graphs and music to enrich the stimulus material. In addition, ERP used in this study records a wide range of brain wave data. More complicated analysis methods might be used to obtain further valuable information. In our research, data analysis was time locked. In the future investigations it would be worth trying some new methods like reaction locked method and traceability analysis. Future studies should also look into the confounding variables more strictly in order to get more refined results.

## Author Contributions

YX: Main research conceptual framework and reporting. MC: Literature collection/review, Data collection. HL: Design and analysis. WZ: Referencing and reporting. ZZ: Methods and formatting. CMA: Refinement of article literature review and methodology, editing of article, formatting and framework corrections, answering reviewers’ comments.

## Conflict of Interest Statement

The authors declare that the research was conducted in the absence of any commercial or financial relationships that could be construed as a potential conflict of interest.
